# Association between cardiovascular risk and maternal perception of BMI in Peruvian schoolchildren

**DOI:** 10.3389/fpubh.2024.1277157

**Published:** 2024-03-20

**Authors:** Margoth Ccari Mamani, Jhosset Flores Martínez, Raquel Chilón Llico, Liset Z. Sairitupa-Sanchez, Sandra B. Morales-García, Oriana Rivera-Lozada, Wilter C. Morales-García

**Affiliations:** ^1^Escuela Profesional de Nutrición Humana, Facultad de Ciencias de la Salud, Universidad Peruana Unión, Lima, Perú; ^2^Escuela Profesional de Psicología, Facultad de Ciencias de la Salud, Universidad Peruana Unión, Lima, Perú; ^3^Departamento Académico de Enfermería, Obstetricia y Farmacia, Facultad de Farmacia y Bioquímica, Universidad Científica del Sur, Lima, Perú; ^4^Facultad de Educación, Universidad Nacional Mayor de San Marcos, Lima, Perú; ^5^Escuela de Medicina Humana, Facultad de Ciencias de la Salud, Universidad Peruana Unión, Lima, Perú; ^6^Escuela de Posgrado, Universidad Peruana Unión, Lima, Perú; ^7^Facultad de Teología, Universidad Peruana Unión, Lima, Perú; ^8^Sociedad Científica de Investigados Adventistas, SOCIA, Universidad Peruana Unión, Lima, Perú

**Keywords:** maternal perception, overweight and obesity, schoolchildren, cardiovascular risk, Peruvian

## Abstract

**Introduction:**

In the modern era, the maternal perception of children’s nutritional status has emerged as a critical area of study, given its potential influence on nutritional interventions and long-term child health. The relationship between this perception and children’s Body Mass Index (BMI) by age is particularly intriguing, as it may reveal discrepancies between perception and reality.

**Objective:**

The aim of this study was to evaluate Peruvian mothers’ perception of their children’s Body Mass Index (BMI) in relation to age and to determine how this perception associates with the children’s cardiovascular risk. The study also analyzed sociodemographic factors that might influence this perception.

**Methods:**

The study included 130 mothers of schoolchildren aged 5 to 11 from a school in Lima. Mothers’ perceptions of their children’s weight were assessed using pictograms, and sociodemographic characteristics were collected through a questionnaire. Weight and height measurements were taken to calculate BMI, and waist circumference was measured to classify cardiovascular risk.

**Results:**

A total of 57.4% of the schoolchildren presented with excess malnutrition, and 51.5% of the mothers incorrectly classified the actual BMI/Age of their children (kappa 0.11; *p* ≤ 0.05). Additionally, it was found that the schoolchild’s age is associated with the mother’s failure to accurately perceive her child’s weight (OR 1.59). Lastly, there was a significant association between maternal perception and cardiovascular risk (*p* ≤ 0.05).

**Conclusion:**

There is a significant discrepancy between maternal perception and the actual nutritional status of children, which can increase cardiovascular risk. It is necessary to implement intervention and education strategies targeted at parents to enhance the recognition and management of childhood overweight and obesity.

## 1 Introduction

Childhood obesity has emerged as a significant public health challenge in the 21st century (1). According to the World Health Organization (WHO), this phenomenon has seen alarming growth, having tripled from 1975 to 2016, leading to approximately 2.8 million annual deaths (2). Lobstein et al. (3) underscores this concern, noting that globally 13% of children are overweight and 11% suffer from obesity (4).

In Latin America and the Caribbean, the situation is equally alarming. It is estimated that overweight affects 30.6% of the population, including 3.9 million preschoolers and 75 million school-aged children (5–7). In Peru, specifically, the National Center for Food and Nutrition (CENAN) reported that during 2017–2018, four out of ten children aged 5 to 9 were overweight, a rate that has doubled in the last decade. Metropolitan Lima, the capital district, showed a concerning 49.3% prevalence in this age group (8).

Childhood obesity is a concern in its own right and is also a predictor of adult obesity, thereby increasing the risk of non-communicable diseases and early adult mortality (9, 10). Factors such as poor dietary habits and sedentary behaviors, largely influenced by the environment and parental habits, contribute to this epidemic (11).

In this context, parental perception, especially that of mothers, regarding their children’s weight plays a critical role. Mothers, traditionally the primary caregivers, significantly influence their children’s dietary habits (12, 13). However, studies have shown that many mothers with overweight or obese children tend to underestimate their weight, thus reducing the likelihood of taking preventive actions and engaging in weight-loss interventions for their children (14–18). This misperception was highlighted by Trejo et al. (19), who found that most Peruvian parents with overweight or obese children did not accurately perceive their children’s weight (19).

To better understand the determinants of this perception, factors associated with inaccurate perception of children’s weight have been studied. Sociodemographic characteristics like the educational level of mothers, ethnic background, and knowledge about healthy eating have been identified as factors linked to misperceptions of children’s weight (19–27). These characteristics, in connection with perceptions of children’s body image, may be crucial in identifying risk groups and developing preventive strategies against childhood overweight and obesity. Hence, this study aims to assess Peruvian mothers’ perception of the Body Mass Index (BMI) in relation to their children’s age and to determine its association with the children’s cardiovascular risk, while also examining the sociodemographic factors that might influence this perception.

### 1.1 Hypothesis

The perception of Peruvian mothers about the Body Mass Index (BMI) of their children in relation to their age is significantly associated with the cardiovascular risk of these children. It is expected that sociodemographic factors influence the accuracy of this maternal perception, thereby contributing to cardiovascular risk in the Peruvian pediatric population.

## 2 Materials and methods

### 2.1 Design and participants

A descriptive, analytical, and cross-sectional study was designed. The sample size was calculated using the G* Power 3.1.9.7 program for an *a priori* analysis. To determine the required sample size for our study, an *a priori* calculation was performed using the G*Power software version 3.1.9.2. A medium effect size (0.3), an alpha significance level of 0.05, and a statistical power of 80% (1-β = 0.80) were established. The necessary sample size calculated was 108 mother–child dyads. Dyads of mothers and children aged 5 to 11 years were included, involving biological mothers and their children who are stable residents in the area. Mothers with chronic illnesses or training in nutrition or health were excluded, as were children with pathologies that prevent anthropometric evaluation. The study ensured diversity in education, nutritional knowledge, and socioeconomic status. Informed consent and availability for follow-up were also required.

### 2.2 Ethical procedures

Initially, the Ethics Committee of a Peruvian university (2212-2022/UPEU-FCS-*CF*) thoroughly reviewed and approved our research protocol. Before the commencement of data collection, an informational session was held with the participating mothers. During this session, the purpose and objectives of the research were thoroughly explained. The importance of their participation was emphasized, and they were assured that their involvement would be entirely voluntary. Once the mothers had a clear understanding of the study, they were presented with an informed consent document. This document outlined the study’s procedures, participants’ rights, and guarantees of confidentiality. Mothers who chose to participate in the study signed this document, thus confirming their understanding and voluntary consent. The mothers were assured that all information collected during the study would be treated confidentially and that both their identities and those of their children would remain anonymous. Strict protocols were established to ensure that data was stored and managed securely and responsibly. This study adhered to the guidelines and principles set forth in the Helsinki Declaration, a set of ethical recommendations and principles for conducting research involving human beings (28).

### 2.3 Variables

#### 2.3.1 Mother’s perception of BMI

The Collins scale (“Collins BMI Measurement Test”) was utilized to assess mothers’ perception of their children’s BMI. Validated in the study “Body Figure Perceptions and Preferences Among Preadolescent Children,” this scale achieved a test–retest reliability level of 0.91 (29). This instrument has been employed in Latin American child populations aged 6 to 10, sharing characteristics similar to our study population (14). The scale categorizes BMI/Age using seven anatomical silhouettes for both males and females, depicting the child’s physical appearance. These silhouettes progress in robustness, aligning with WHO classification parameters. Each silhouette is associated with a BMI value, ranging from 12.1 kg/m^2^ to 35.5 kg/m^2^: silhouettes 1–2 signify underweight; 3–5, normal weight; 6, overweight; and 7, obesity. These figures did not exhibit the specified BMI values when presented. Mothers were asked to select the silhouette that, in their perception, resembled their child’s body image, using the question, “Which image identifies your child?” The perceived BMI values were then compared with the actual BMI derived from weight and height measurements. This comparison facilitated an analysis of whether there is an accurate or inaccurate perception of the child’s actual weight.

#### 2.3.2 Mother’s sociodemographic characteristics questionnaire

Data was collected regarding the mother’s age, her child’s age, educational level (classified as: “no studies,” “primary,” “secondary,” and “higher education”), and birth region (classified as: “coast,” “mountains,” and “jungle”).

#### 2.3.3 Mother’s knowledge of healthy eating

The questionnaire “Knowledge about healthy food among Peruvian public university students” was used to determine mothers’ knowledge of healthy eating. Based on a literature review of nutrition and the WHO’s nutritional pyramid, the questionnaire includes 16 questions on healthy eating knowledge, each correct answer scoring 2 points. Maternal knowledge was categorized into three levels: <17 points indicating low knowledge; 17 to 25 points as medium knowledge; and > 25 points as high knowledge. It has a Kuder Richardson reliability coefficient of 80.7%, validating its application (30).

#### 2.3.4 Anthropometric measurements

Two professional nutritionists conducted anthropometric measurements. Weight was measured using a SECA 750 floor mechanical scale, calibrated to zero and with a capacity of 150 kg. Measurements were taken with the child in light clothing and barefoot. Height was measured using a CENAN-standardized wooden stadiometer, capable of measuring up to 199 cm. The child was positioned with their head according to the Frankfurt plane, heels together at the lower end, and feet angled between 45 to 60 degrees.

#### 2.3.5 Body mass index

The BMI/Age diagnosis followed the World Health Organization’s criteria for children aged 5 to 19 years (31). Underweight is defined as a value less than or equal to −2 SD (Standard Deviations), normal weight as greater than -2SD and less than or equal to +1SD, overweight as greater than +1SD and less than or equal to +2SD, and obesity as greater than +2SD and less than or equal to +3SD.

#### 2.3.6 Abdominal circumference

A self-retracting LUFKIN steel tape measure, with a range up to 200 cm and a resolution of 1 mm, was used to determine abdominal circumference. The abdominal circumference was categorized by sex and age (AC/A) into low cardiovascular risk (< p75), high cardiovascular risk (≥ p75 and < p90), and very high cardiovascular risk (≥ p90) (32).

### 2.4 Statistical analysis

A correlation was established between maternal perception of their children’s BMI/Age and associated sociodemographic factors. Additionally, the association between maternal perception and cardiovascular risk was identified. The collected data were analyzed using SPSS version 26.0, with a focus on the study’s objectives. Quantitative variables were described using measures of central tendency and dispersion, and qualitative variables were presented with absolute frequency and percentage. The concordance between maternal perception and their children’s BMI/Age was determined using the Kappa coefficient (*p* ≤ 0.05). Bivariate logistic regression analysis was employed to evaluate sociodemographic factors associated with maternal perception. The relationship between maternal perception and cardiovascular risk, diagnosed by abdominal circumference, was examined using the non-parametric Pearson chi-square test (p ≤ 0.05), and both variables were also correlated using Cramer’s V coefficient.

## 3 Results

### 3.1 Participant characteristics

Table 1 details the characteristics of schoolchildren (*n* = 130) and their mothers (*n* = 130) in Lima, Peru, in 2023. The children were aged between 5 to 11 years (*M* = 8, SD = 1.8), with a majority being female (51.5%). The average Body Mass Index (BMI) was 19.4 kg/m^2^ ± 3.7, with 43.1% classified as normal weight, 30.8% overweight, and 26.2% obese. The average abdominal circumference was 67.4 cm ±9.7. In terms of cardiovascular risk based on abdominal circumference, 46% were at low risk, 23% at high risk, and 31% at very high risk. Regarding the mothers, their age range was between 20 to 60 years (*M* = 29.5, SD = 7.4). The majority of the mothers were between 25 to 60 years old (78%), came from the coastal region (60%), had a higher education level (85%), and possessed a medium level of knowledge about healthy eating (71%).

**Table 1:** Descriptive characteristics of schoolchildren and mothers (*n* = 130), Lima – Peru, 2023.

Schoolchildren characteristics	*M* (SD)/*n* (%)
Age	8 ± (1.8)
Child’s BMI	19.4 ± (3.7)
Abdominal circumference	67.47 ± (9.7)
Gender	
Male	63 (48.5%)
Female	67 (51.5%)
Children’s nutritional status (BMI)	
Normal	56 (43.1%)
Overweight	40 (30.8%)
Obesity	34 (26.2%)
Cardiovascular risk diagnosis (AC)	
Low risk	60 (46%)
High risk	30 (23%)
Very high risk	40 (31%)

Mothers’ characteristics	*M* (SD)/*n* (%)
Age	29.5 ± (7.4)
Age range	
20 to 25 years	27 (21%)
25 to 60 years	101 (78%)
Over 60 years	2 (1%)
Region of birth	
Coast	78 (60%)
Highlands	48 (37%)
Jungle	4 (3%)
Mother’s education	
Secondary school	20 (15%)
Higher education	110 (85%)
Knowledge level about healthy eating	
Low knowledge	20 (15%)
Medium knowledge	92 (71%)
High knowledge	18 (14%)

BMI, Body Mass Index; AC, Abdominal Circumference; M, Mean; SD, Standard Deviation; n: Participants; %: Proportion.

### 3.2 Preliminary analysis

When associating maternal perception with the actual BMI/Age diagnosis of their children (Table 2), it was observed that 48.5% of mothers accurately perceived their children’s weight status with a kappa coefficient of 0.119 (minimal agreement) (*p* = <0.05). Of these, those with normal weight were correctly classified in 43.1% (*n* = 56) of cases, and those with overweight in 5.4% (*n* = 7) of cases. Of the group of mothers (51.6%) who had an inaccurate perception, 25.4% (*n* = 33) underestimated their overweight children as normal weight; while mothers with obese children underestimated them as “normal weight” in 18.5% (*n* = 24) and “overweight” in 7.7% (*n* = 10) of cases.

**Table 2:** Maternal perception of BMI of their children (Ages = 5–11 years, *n* = 130), Lima – Peru, 2023.

Maternal perception	Child’s BMI								*p*-value Kappa
	Normal		Overweight		Obesity		Total		
	*n*	%	*n*	%	*n*	%	n	%	
Normal	56	43.10%	33	25.40%	24	18.50%	113	86.90%	*p* < 0.05
Overweight	0	0%	7	5.40%	10	7.70%	17	13.10%	*K* = 0.119
Obesity	0	0%	0	0%	0	0%	0	0%	
Total	56	43.10%	40	30.80%	34	26.20%	130	100%	

*p* < 0.05, K, Kappa value.

### 3.3 Sociodemographic factors and maternal perception of child weight

Table 3 displays the sociodemographic characteristics of the 130 surveyed mothers. Of these, 84.6% had higher education and the remaining 15.4% secondary education. Regarding birth region, 60% of mothers were from the coast, 37% from the mountains, and 3% from the jungle. In terms of maternal knowledge about healthy eating, 72% demonstrated medium knowledge, while 14% were classified with high knowledge. Regression analysis between variables indicated that education level, birth region, and knowledge about healthy eating did not significantly correlate with maternal perception (*p* > 0.05). However, it was found that the child’s age from 5 to 11 years (OR 1.59) significantly affected the mother’s inaccurate perception of her child’s weight (*p* < 0.05).

**Table 3:** Bivariate analysis of sociodemographic factors and maternal perception of child’s weight (*n* = 130), Lima – Peru, 2023.

Variables	Descriptive analysis		Bivariate analysis		
	Maternal perception		Odds ratio	IC 95%	*p*-value
Child’s age	5 years – 11 years (*M* = 8. SD = 1.8)		1.59	0.086–0.358	0.004
Education level	Adequate	Inadequate			
Secondary	5.40%	9.20%	0.49	0.166–1.468	0.575
Higher	41.50%	43.10%	–	–	
*Birth region*					
Coast	28.50%	31.50%	1.07	0.139–8.285	
Highlands	16.90%	20.00%	0.87	0.106–7.155	0.991
Jungle	1.50%	1.50%	–	–	
*Knowledge level about healthy eating*					
Low knowledge	6.90%	7.70%	1.39	0.346–5.611	
Medium knowledge	33.10%	38.50%	1.68	0.531–5.334	0.667
High Knowledge	6.90%	6.90%	–	–	

95% CI: 95% confidence interval.

### 3.4 Maternal perception of BMI and its association with cardiovascular risk in children

Table 4 shows a significant association between maternal perception of their children’s Body Mass Index (BMI) and the classification of cardiovascular risk based on abdominal circumference. Adequate maternal perception predominantly correlated with low risk (36.2%), while inadequate perception was notably linked with very high cardiovascular risk (27.7%). Additionally, 15.4% of inadequate perceptions were associated with high risk. The statistical significance of the association (p < 0.05) and a Cramer’s V coefficient of 0.607 indicate a strong correlation between mothers’ perception of their children’s weight and the actual classification of cardiovascular risk.

**Table 4:** Association between inadequate maternal perception of child’s BMI and cardiovascular risk (*n* = 130), Lima – Peru, 2023.

Maternal perception	Cardiovascular risk classification (abdominal circumference)								*p*-value
	Low risk		High risk		Very high risk		Total		
	*n*	%	*n*	%	*n*	%	*n*	%	
Adequate	47	36.2%	10	7.7%	4	3.1%	61	46.9%	
Inadequate	13	10.0%	20	15.4%	36	27.7%	69	53.1%	<0.05
Total	60	46.2%	30	23.1%	40	30.8%	130	100%	

V Cramer = 0.607.

## 4 Discussion

The epidemic of childhood obesity, with its association with non-communicable diseases and increased mortality, is particularly concerning in Peru. Over the last decade, the rates of overweight children have doubled, especially in Metropolitan Lima (33, 34). Maternal perception of their children’s weight is critical, as mothers play a key role in shaping family eating habits. Studies indicate that many mothers underestimate the weight of their overweight or obese children (19). Additionally, sociodemographic factors, such as the mother’s educational level and knowledge about healthy eating, have been linked to inaccurate perceptions of child weight (21, 24). This study assesses Peruvian mothers’ perception of their children’s BMI and its relation to cardiovascular risk, hypothesizing that this perception significantly associates with cardiovascular risk in children and is influenced by sociodemographic factors, underscoring the importance of addressing this perception for effective preventive strategies.

The current study addressed maternal perception of their children’s BMI/Age compared to the actual BMI/Age, revealing a significant discrepancy between the two. No association was found between the mother’s sociodemographic factors (such as education, birth region, and knowledge about healthy eating) and perception of their children’s BMI. However, a significant relationship was identified when considering the child’s age. Additionally, a strong correlation was established between maternal perception and the children’s cardiovascular risk, assessed through abdominal circumference. These findings underscore the complexity of parental perception regarding their children’s nutritional status. The discrepancies observed between different studies can be attributed to varied methodologies used to assess parental perception, whether verbal or visual (18, 22, 35). However, it’s crucial to consider ongoing cultural and social changes. In contemporary society, significant epidemiological and dietary transitions are occurring. In this context, what was previously classified as overweight in children may now be perceived as “normal weight” by family and society. The rising prevalence of childhood obesity has desensitized society to excess weight, normalizing this condition to some extent. Furthermore, a lack of awareness may lead parents to base their perceptions on visual comparisons with other children, who may also be overweight (36). Parental perception is complex, influenced by various factors including the relationship between the observer and the observed, the individual characteristics of both, and the observer’s prior beliefs and experiences (37).

Maternal perception of their children’s nutritional status, especially concerning BMI/Age, is an increasingly relevant topic in nutritional research. The present study identified a significant association between the child’s age and the mother’s inaccurate perception of BMI/Age. Specifically, within the 5 to 11 year age range, there is a tendency for mothers to misperceive their children’s actual BMI/Age. Although no direct comparisons with children outside this age range were made, these findings align with existing literature suggesting that age influences parental perception of nutritional status. Research by Ramirez et al. (16) and Zhang et al. (38) reported challenges in maternal perception mainly in ages between 4 and 9 years. Conversely, studies like that of AlHasan et al. (20) suggest that identifying overweight and obesity is more challenging in younger children, aged 2 to 4 years. Cultural beliefs associating early weight gain with health and well-being, and expectations of outgrowing overweight with growth and increased physical activity, may contribute to these perception differences, as discussed by Alshahrani et al. (37) and Esteban-Vasallo et al. (39).

The study revealed a concerning discrepancy between maternal perception of their children’s nutritional status and the actual cardiovascular risk they face. Notably, 43.1% of overweight or obese children, not correctly identified by their mothers, exhibited a high and very high risk of developing cardiovascular diseases based on abdominal circumference. Conversely, only 10.8% of children whose nutritional status was accurately perceived by their mothers presented a high or very high cardiovascular risk. These findings emphasize the importance of parental perception in identifying and managing cardiovascular risk in children. Although most studies on maternal perception have focused on BMI/Age as the primary indicator, other markers are crucial. A recent study supports this, noting that parental underestimation of children’s weight status directly correlates with a higher risk of cardiovascular disease (40). Abdominal circumference emerges as a critical anthropometric indicator. Unlike BMI/Age, which measures excess weight relative to height, abdominal circumference directly measures central adiposity, a factor closely associated with increased cardiovascular disease risk. BMI/Age has limited sensitivity in detecting excess adiposity, possibly leading to underestimations of the actual risk (41). The implications of these findings are significant, considering the progression of cardiovascular diseases. As noted by Santos et al. (42), cardiovascular risk can begin in early childhood and persist asymptomatically until adulthood.

### 4.1 Implications

The discrepancy between maternal perception and the actual nutritional status of children underscores the importance of integrating nutritional education into Peru’s academic curriculum. This integration serves as a strategy to enhance knowledge about healthy habits. Health professionals need to be cognizant of this perceptual gap and should focus on improving communication and education directed toward parents and caregivers. These stakeholders require effective resources to recognize and manage childhood overweight and obesity. The development of public policies is crucial to strengthen nutritional education and raise awareness about childhood obesity. This includes campaigns that address misconceptions and foster a clear understanding of associated risks. Additionally, the inclusion of anthropometric measures such as abdominal circumference in routine health check-ups for children is worth considering. From a theoretical perspective, it is essential to investigate how cultural beliefs and social norms influence parental perception. Understanding these influences is key to identifying barriers and facilitators in combating childhood obesity.

### 4.2 Limitations

This study contributes to a limited body of research on maternal perception related to BMI/Age and abdominal circumference in Peruvian schoolchildren. However, several significant limitations should be noted: Firstly, the study was conducted among schoolchildren and their mothers residing in an urban area, which may explain the sample’s homogeneity, characterized by generally high maternal education and moderate maternal knowledge about healthy eating. Secondly, the sample was collected from an urban school in Lima through convenience sampling, and therefore might not be representative, limiting the generalizability of the current findings. It’s essential to recognize that our study has limitations that could impact the interpretation of our findings. The cross-sectional nature of our study precludes the establishment of causal relationships between maternal perception and the actual nutritional status of children. Although significant associations were identified, we cannot conclusively assert that inaccurate maternal perception directly leads to higher cardiovascular risk in children. Longitudinal studies are recommended for more precise insights. Additionally, our study focused mainly on maternal perception, neglecting the perspectives of other caregivers, such as fathers, grandparents, or guardians. Each caregiver, with their unique cultural and experiential background, could offer a distinct viewpoint on the child’s nutritional status. Excluding these caregivers may have led to overlooking important aspects of parental perception. Lastly, while we considered some sociodemographic variables, there are other factors that might influence maternal perception, including cultural beliefs, media exposure, and mothers’ personal experiences with weight and nutrition.

### 4.3 Conclusion

In conclusion, this study reveals a discrepancy between mothers’ perceptions and the actual nutritional status of their children, indicating a tendency to underestimate weight in cases of overweight or obesity, which may contribute to increased cardiovascular risk. These findings highlight the necessity of directing nutritional interventions toward parents of school-aged children, especially at critical ages where maternal perception is likely to be less accurate. For public health, this study recommends implementing educational programs that enhance parents’ ability to recognize and address overweight and obesity, as well as advocating for policies that promote greater awareness and prevention of cardiovascular risk from childhood. It is vital for intervention strategies to assess the impact of maternal perceptions and their influence on family health behaviors.

## Data availability statement

The raw data supporting the conclusions of this article will be made available by the authors, without undue reservation.

## Ethics statement

The research involving humans was approved by the Ethics Committee of the Peruvian Union University (2212-2022/UPEU-FCS-CF). The studies were conducted in accordance with local legislation and institutional requirements. The legal guardians or closest relatives of the participants provided written informed consent for participation in this study.

## Author contributions

MM: Conceptualization, Data curation, Formal analysis, Supervision, Validation, Visualization, Writing – original draft, Writing – review & editing. JM: Resources, Software, Supervision, Validation, Visualization, Writing – original draft, Writing – review & editing. RL: Formal analysis, Funding acquisition, Investigation, Methodology, Project administration, Writing – original draft, Writing – review & editing. LS-S: Conceptualization, Data curation, Formal analysis, Funding acquisition, Software, Supervision, Writing – original draft, Writing – review & editing. SBM-G: Methodology, Validation, Visualization, Software, Writing – review & editing. OR-L: Conceptualization, Data curation, Formal analysis, Funding acquisition, Resources, Software, Supervision, Visualization, Writing – original draft, Writing – review & editing. WM-G: Conceptualization, Data curation, Methodology, Project administration, Software, Supervision, Validation, Visualization, Writing – original draft, Writing – review & editing.
